# Odors Adsorption in Zeolites Including Natural Clinoptilolite: Theoretical and Experimental Studies

**DOI:** 10.3390/ma17133088

**Published:** 2024-06-24

**Authors:** Izabela Czekaj, Natalia Sobuś

**Affiliations:** 1 Zeocomplex, Płk. Stanisława Dąbka 17, 30-832 Kraków, Poland; info@zeocomplex.com; 2 Faculty of Chemical Engineering and Technology, Cracow University of Technology, Warszawska 24, 31-155 Kraków, Poland

**Keywords:** odors adsorption, ammonia, carboxylic acids, dft, zeolite, clinoptilolite

## Abstract

This publication presents the results of combined theoretical and experimental research for the potential use of natural clinoptilolite zeolite (CLI) as an odor-adsorbing material. In this study of adsorption capacity, CLI of various granulation was used and its modifications were made by ion exchange using Sn and Fe metals to check whether the presence of metals as potential active centers does not lead to catalytic processes and may lead to enhanced absorption of odorous substances through their adsorption on the created metallic forms. Additionally, in order to increase the specific surface area, modifications were made in the form of hierarchization in an acidic environment using hydrochloric acid to also create the hydrogen form of zeolite and thus also check how the material behaves as an adsorbent. To compare the effect of CLI as a sorption material, synthetic zeolite MFI was also used—as a sodium form and after the introduction of metals (Sn, Fe). The above materials were subjected to adsorption measurements using odorous substances (including acetaldehyde, dimethylamine, pentanoic acid and octanoic acid). Based on the measurements performed, the most advantageous material that traps odorants is a natural material—clinoptilolite. Depending on the faction, its ability varies for different compounds. In the case of acetaldehyde, an effective material is clinoptilolite with a grain size of up to 2 mm. In the case of carboxylic acids, it is material after hierarchization with a fraction of 3–4 mm. In the case of theoretical calculations, information was obtained to show that metallic centers are more stable above oxygen, which is associated with the skeletal aluminum in clinoptilolite.

## 1. Introduction

Zeolites are a group of hydrated aluminosilicates with a microporous structure. They are characterized by a large specific surface area and ion-exchange ability. These materials have been used as membranes, adsorbents, ion exchangers, catalysts or molecular sieves for the treatment of industrial and municipal pollution [1].

Aluminosilicates include zeolites of synthetic and natural origin. Deposits of natural zeolites, including clinoptilolite, are common in many countries such as Greece, Italy, the United Kingdom, Mexico and Turkey. The properties of zeolites in natural materials mimic the properties of many of their synthetic counterparts. Due to their structure, zeolites have many applications, from being used as fillers to molecular sieves and ion exchangers. In recent years, natural zeolites have been studied for their use as catalysts. However, they still require thorough analysis and modification, as they have enormous potential for use on a larger scale [2].

Clinoptilolite minerals suitably modified show selectivity towards cations, anions and can be used as a material for the removal of contaminants. Many studies have been performed on the activation of clinoptilolite by heat treatment or acid treatment, resulting in the elimination of cations from the zeolite structure and pores, obtaining H-zeolite forms and partial degradation of the crystal structure (dealumination). On this basis, two possible modifications of natural zeolite were developed: (i) conversion to the ammonium form of zeolite and heat treatment and (ii) direct ion exchange in dilute acid solution. Activated natural zeolite was used as an adsorbent for the removal of contaminants and was shown to be more effective than activated carbon [3,4]. In addition, the presence of metals such as Zn and Ag makes it possible to achieve an efficiency of 90% during water purification. In the work of Russo and co-authors [5], an adsorption process using natural zeolite (clinoptilolite) modified with iron was carried out for the removal of BTX compounds (benzene, toluene, xylene) from groundwater. Preliminary studies conducted suggest a promising application of iron-modified clinoptilolite. The combination of adsorption and catalytic oxidation enabled the removal of organic BTX contaminants (15 mg) from a pH-neutral aqueous solution without releasing iron ions from the zeolite structure. The adsorption capacity of clinoptilolite in its activated form during the removal of organic compounds (phenol, chlorophenol) from wastewater was studied. The adsorption rate of aromatic compounds was determined to be fast, and the adsorption equilibrium was reached after 45 min. The adsorption of phenol and chlorophenol was calculated to be 7.977 mg/g for phenol, 9.846 mg/g for o-chlorophenol, 9.981 mg/g for m-chlorophenol and 8.241 mg/g for p-chlorophenol, respectively [6]. Natural zeolite has also been used as an adsorption material to remove the unpleasant odors of ammonia and hydrogen sulfide. For this purpose, first of all, the material was appropriately activated by chemical modification—treatment with acid (HCl 0.4 M for 2 h) and base (NaOH 1 N for 2 h) and thermal treatment (300 °C for 3 h). Subsequently, tests were carried out in which the best material for the adsorption of ammonia and hydrosulfuric acid turned out to be zeolite with a grain diameter of 1–3 mm, activated by thermal treatment [7].

### 1.1. Odorous Substances

This discussion focuses on two industries, catering and breeding (on the example of pig farms), and analyzes the composition of odor mixtures in both these industries, followed by a comparative analysis of the chemical structure of substances and particle size, which are important for the adsorption process in porous materials such as zeolites.

#### 1.1.1. Odors in Catering

The catering processes, where the products are quickly processed at high temperatures, can be a source of organic compounds including n-alkanes, polycyclic aromatic hydrocarbons (PAHs), fatty acids, as well as aromatic amines (AAs) and the already mentioned heterocyclic aromatic amines (HAAs) [8,9,10]. Of these, formaldehyde, acetaldehyde, acrolein, dimethylamine and volatile organic compounds (VOCs) including acetone, toluene or octane appear to be the most significant (Table 1) [10].

**Figure 1:**
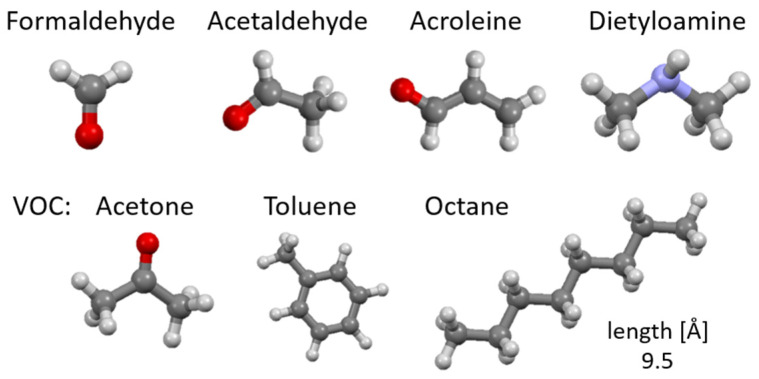
Chemical structure of odorous compounds found in catering (our work). Elements color: red—oxygen, light blue—nitrogen, dark gray—carbon, lighter gray—hydrogen.

Figure 1 shows examples of the chemical structures of compounds that may be odorous substances and are formed during cooking or are present in animal excrements. Most of the compounds presented have a negative impact on the health and life of living organisms; hence, there is a need for their presence to be negligible. Table 1 shows where they are most often present and what their maximum permissible concentrations are so that they do not pose a threat to living organisms.

#### 1.1.2. Odors in Breeding

There are many chemical substances, including odorous ones, present in poultry farms and livestock farms. Hydrogen sulfide and ammonia were most frequently observed. Other compounds are the group of thiols and sulfides. There are also aromatic compounds, such as aromatic amines, containing sulfur and nitrogen in their structure, toulene or xylene and a wide range of chemical compounds such as aldehydes, ketones and esters [11].

**Figure 2:**
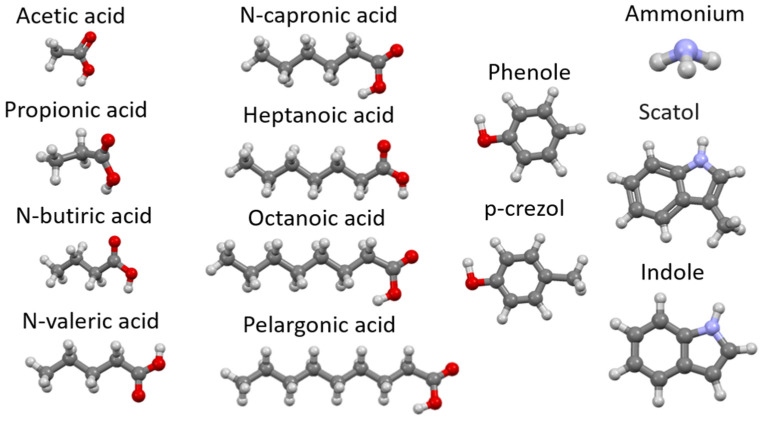
Chemical structure of odorous compounds found in piggery pollutants—molecules are given (relevant due to diffusion into zeolite pores during adsorption) (our work). Elements color: red—oxygen, light blue—nitrogen, dark gray—carbon, lighter gray—hydrogen.

Table 2 presents average of vent gas pollutant concentrations from piggery [11] in the ventilation conditions to ensure an appropriate climate for the development of the animals, exchanging 1 m^3^ of air for 1 h and 1 kg of adult livestock (Figure 2). Based on Table 2, it can be seen that in the case of adult pigs, the most unpleasant odorous chemical emitted in breeding is ammonia. Its concentration in the tests performed is 18 mg/m^3^. The next compounds are substances classified as organic acids, where the highest concentration was observed for acetic acid (6.7 mg/m^3^) and isovaleric acid (0.21 mg/m^3^). The above-mentioned carboxylic acids have characteristic odors and, together with ammonia, may create unpleasant smelling and harmful mixtures. You can also notice the presence of skatoles and indoles, which in small concentrations are unpleasant to smell and toxic to animals and humans. Numerous odors are mentioned in this work, which show the high similarity of adsorption inside the tested zeolites due to chemical similarity or presence of functional groups adsorbing in a similar way. For example, ammonia and dimethylamine adsorb on the same adsorption centers, as do amines (methylamine, indole) or thiols. So, in order to obtain the best information, dimethylamine was selected for laboratory work (which allowed for the faster selection of zeolites with the best adsorption properties and more efficient testing of their appropriate granulation) [11].

It is the same for numerous organic acids, from which short-chain acids (propionic acid and pentanoic = valeric acid—to study the adsorption process via the -COOH group) and long-chain acids (octanoic acid—to study the steric valve due to the length of carbon chain) were selected [11].

Based on the literature review, one can notice interest in the topic of adsorption using zeolites and the analysis of odorous and toxic chemical compounds present in animal farms. Therefore, this work presents attempts to test the adsorption capacity of zeolites with selected chemical compounds. In the experimental part, natural zeolite was used and its sorption properties were compared with the use of a synthetic hydrogen form of MFI. The research also includes modification of zeolites in the form of CLI hierarchization and ion exchange with the incorporation of tin and iron for both materials. The research also used material without CLI modification. Additionally, molecular modeling and calculations were also performed for the natural form of zeolite to compare and confirm the ability of clinoptilolite to adsorb chemical compounds.

## 2. Materials and Methods

### 2.1. Zeolites Preparation

In the study, natural zeolite—clinoptilolite (VSK PRO-ZEO, Vranov nad Topľou, Slovakia)—and synthetic MFI (Module 85.7, Clariant, Munich, Germany) zeolite were used as adsorption materials. In the case of natural zeolite, various granulations were used (up to 0.2 mm, 1–2 mm and 3–4 mm) and the preparation methods were grinding and crushing. In the case of synthetic, grain sizes up to 0.2 mm were used.

Natural zeolite was modified by ammonium exchange. An amount of 2.5 g of clinoptilolite was weighed accordingly and a 0.5 mol/L solution of NH_4_NO_3_ (Merck KGaA, Darmstadt, Germany) was prepared (1 g solid state to 100 mL salt solution). The ammonium exchange was carried out for 4 h at the temperature of 80 °C. After that, the cooled suspension was washed several times with deionized water, centrifuged and dried for 1 h at 100 °C and calcined for 5 h at 450 °C. The ammonium exchange was performed twice using the grinding and crushing forms with 0.2 mm size. Samples were coded as ground-NH_4_-CLI and crushed-NH_4_-CLI.

Zeolites after ammonium exchange were further modified by ion exchange and the introduction of metals. For this purpose, aqueous solutions of appropriate metal salts were prepared with a concentration of 0.25 mol/L—SnCl_4_∙5H_2_O and Fe(NO_3_)_3_∙9H_2_O (1 g solid state to 100 mL salt solution) (Merck KGaA, Darmstadt, Germany). Ion exchange was carried out at 80 °C for 24 h. After that, the suspension was washed several times with deionized water, centrifuged and dried for 1 h at 100 °C. The dried samples were calcined at 450 °C for 5 h in order to remove impurities. The obtained samples were named: ground-Sn-CLI, crushed-Sn-CLI and ground-Fe-CLI, crushed-Fe-CLI. The same modification procedure (process conditions are the same as for natural zeolite) was carried out for the synthetic material, MFI. The material after ammonium exchange was determined as NH_4_-MFI. After the introduction of metals, the samples were named as Sn-MFI and Fe-MFI.

In order to shorten the time of clinoptilolite modification with metals and to compare the sorption capacity of these materials with those obtained above, an ion exchange was performed under hydrothermal conditions, excluding ammonium exchange. For this purpose, 2.5 g of clinoptilolite was used as a grinding and crushing form with 0.2 mm size. Aqueous solutions of appropriate metal salts with a concentration of 0.25 mol/L SnCl_4_∙5H_2_O and Fe(NO_3_)_3_∙9H_2_O were prepared. The ion exchange was carried out for 24 h at 80 °C. After that, the suspension was washed several times with deionized water, centrifuged and dried for 1 h at 100 °C. The dried samples were calcined at 450 °C for 5 h in order to remove impurities. The obtained samples were named: ground-Sn-CLI-WB, crushed-Sn-CLI-WB and ground-Fe-CLI-WB, crushed-Fe-CLI-WB. A similar procedure was performed for synthetic material. The modified samples were named, respectively, Sn-MFI-WB and Fe-MFI-WB.

The process of hierarchization of natural zeolite was also carried out through partial degradation of the crystal structure and the formation of acid active centers. Crushed clinoptilolite with fractions of 0–0.2 mm, 1–2 mm and 3–4 mm was prioritized. For this purpose, 2.5 g of the appropriate zeolite fraction was weighed and then a 2 mol/L aqueous hydrochloric acid solution was prepared. The hierarchization process was carried out for 1 h at 60 °C. Then, the suspension was centrifuged and washed several times with deionized water until pH = 7. The modified zeolite was then dried for 1 h at 100 °C and calcined to remove impurities for 5 h at 450 °C. Samples treated with hydrochloric acid were determined as follows: crushed-CLI-HCl, CLI_1–2 mm-HCl and CLI_3–4 mm-HCl.

### 2.2. Characterization

Powder X-ray diffraction (XRD) for selected materials was performed using a PANalytical X’Pert PRO MPD (Malvern, UK) diffractometer equipped with a CuKα radiation source, at a voltage of 40 kV and an intensity of 30 mA. Scanning was performed in a continuous mode in the range of 2θ from 5 to 55°.

Analysis of FT-IR was performed using infrared spectroscopy using the attenuated total infrared reflection technique. The spectra for selected samples were recorded using FTIR Thermo Scientific Nicolet iS5 (Waltham, MA, USA), equipped with the iD7 ATR accessory in the range of 4000–400 cm^−1^.

Nitrogen sorption at a low temperature was performed using the Micromeritics ASAP 2020 analyzer (Norcross, GA, USA). The specific surface area was determined using the Brunauer–Emmett–Teller (BET) method in the pressure range p/p_0_ = 0.05–0.15. The pore size was determined by the Barrett–Joyner–Halenda (BJH) method with the volume of adsorbed nitrogen as p/p_0_ = 0.98.

The materials were also analyzed using a Thermo ScientificTM Evolution 220 UV-Vis spectrophotometer in diffuse reflectance (DRS) mode. The analysis was performed using a baseline using BaSO_4_ and in the wavelength range of 200–800 nm. Integration time is 0.05 s and scanning speed is 1200 nm/min with a step of 1 nm.

For elemental analysis, spot analysis was performed using an Energy Dispersive X-Ray Spectroscopy (EDS) detector equipped with a HITACHI^®^TM3000 (Tokyo, Japan) scanning electron microscope (SEM) with a beam voltage of 5.15 keV, magnification up to 30,000 times and a resolution of approx. 30 nm.

## 3. Results and Discussion

### 3.1. Characteristics of Zeolite Materials

**Figure 3:**
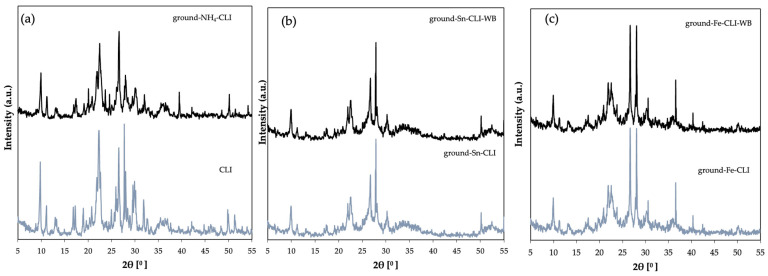
Diffractogram of clinoptilolite: (**a**) natural form (CLI) and ground after ammonium exchange (ground-NH_4_-CLI), (**b**) with incorporated Sn metal, (**c**) with incorporated Fe metal.

Based on the collected diffractograms (Figure 3), it can be seen that the modified samples were not degraded. The initial sample is characteristic of the clinoptilolite material and confirms its use. In addition to the phases derived from clinoptilolite, the natural material contains an admixture of other phases, such as quartz (2θ = 21, 26°) or illite (2θ = 9, 20°) [12]. Ammonium exchange led to a reduction in phase intensity and it can be seen that additional impurities were removed from the material. Further modification of the zeolite leads to partial amorphization of the sample without its degradation, which may confirm that the material has undergone modification in the form of ion exchange. Another reason may also be that the layered structure of clinoptilolite after ion exchange leads to them moving apart and the distance between the layers increases due to the presence of metals. According to the available literature, reflections from metals for tin as SnO_2_ can be observed at 2θ = 31° [13], and for iron metal in the form of Fe_2_O_3_ at 2θ = 40–45°. In turn, the high peak at 2θ = 26° comes from SiO_2_ [14].

**Figure 4:**
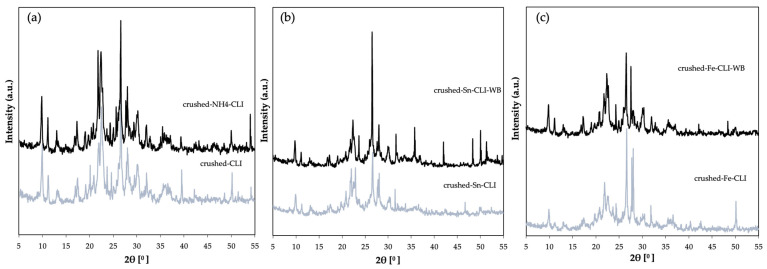
Diffractogram of clinoptilolite: (**a**) natural form (CLI) and crushed after ammonium exchange (ground-NH_4_-CLI), (**b**) with incorporated Sn metal, (**c**) with incorporated Fe metal.

XRD analysis was also performed for a sample—clinoptilolite, which was crushed to a grain size of 0.2 mm (Figure 4). In addition to peaks from clinoptilolite, it can also see quartz and illite. However, the majority of peaks that originate from clinoptilolite dominate. In the case of the material after ammonium exchange, it can be seen that the quartz phase (2θ = 21, 26°) has decreased, which may indicate that it has been washed out. However, looking at both diffractograms before and after ammonium exchange, no significant differences are visible. In the case of ion exchange with tin (Figure 4b) and iron (Figure 4c) using ammonium exchange, there is a significant difference when compared to the starting material. The diffractograms show a typical clinoptilolite phase with no other phases. Additionally, there is a decrease in the intensity of the peaks, which may indicate that the distance between the clinoptilolite layers has increased, as this material has a layered structure [12,13,14].

**Figure 5:**
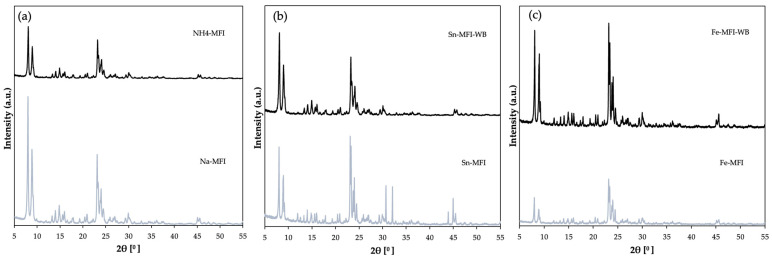
Diffractogram of MFI zeolite: (**a**) sodium form and after ammonium exchange, (**b**) with incorporated Sn metal, (**c**) with incorporated Fe metal.

The above diffractograms (Figure 5) show the structure characteristic of MFI zeolite. It can be seen, in the case of ammonium exchange, that the intensity of the material after modification decreased significantly compared to the starting material. This proves that ammonium exchange and sodium removal took place without degradation of the zeolite crystal structure. Changes in diffractograms after modification with metals (Figure 5b,c) can also be noticed. In the case of ion exchange without prior ammonium exchange, i.e., the direct replacement of sodium with tin or iron metals, this peak intensity is much higher than in the case of two-stage modification of the starting zeolite, which can also confirm that there has been interference in the crystal structure and modification in the material along with partial obtaining of amorphous forms.

**Figure 6:**
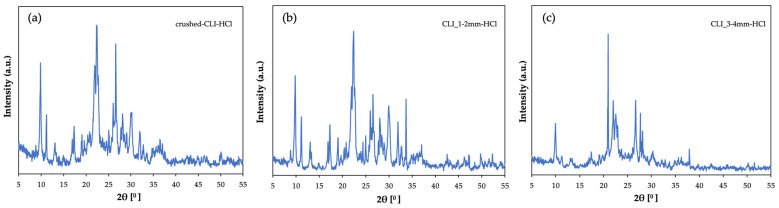
Diffractogram of clinoptilolite zeolite: (**a**) crushed form after hierarchization with HCl (**b**) size 1–2 mm after hierarchization with HCl, (**c**) size 3–4 mm after hierarchization with HCl.

Above are the diffractograms of samples (Figure 6) containing natural zeolite after hierarchization in an acidic environment. For sorption tests, unmodified clinoptilolite with a fraction of up to 2 mm, previously crushed, a 1–2 mm form and a 3–4 mm form were used. The materials were successively treated with 1 mol/L hydrochloric acid for 1 h at 60 °C. Comparing the materials after modification, a decrease in the intensity of peaks coming from clinoptilolite can be seen. This may indicate degradation of the crystalline structure of the material as a result of the action of the acid. This confirms the hierarchy process, regardless of the preparation and diameter of clinoptilolite grains. You can also see the predominance of quartz in the material at 21° [12]. The proposed hierarchization method interferes with the zeolite structure but does not completely degrade it.

**Figure 7:**
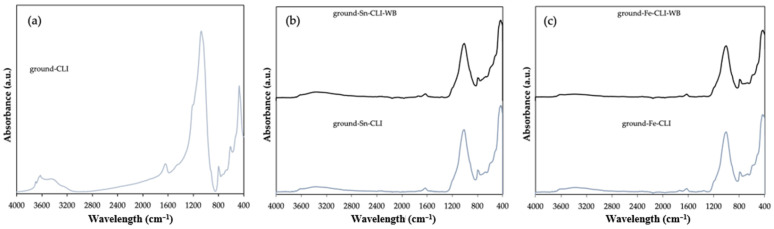
ATR FTIR spectra of ground clinoptilolite without modification (**a**) and after modification with Sn metal (**b**) and Fe metal (**c**).

ATR FT-IR analysis was performed for selected materials. In this case, the starting material is clinoptilolite, which has been ground (Figure 7). Additionally, CLI samples after metal modification with and without ammonium exchange were compared. In the first case, for ground-CLI, one can see a band at wavelengths in the range of 3700–3200 cm^−1^, 1650 cm^−1^ and an intense band at wavelengths 1250–850 cm^−1^. In the case of the band extending from 3700 to 3200 cm^−1^ and at a length of 1650 cm^−1^, these are the bands originating from the hydroxyl group -OH and the band originating from the presence of the so-called zeolite water. In turn, the intense band extending in the wavelength range from 1250 to 850 cm^−1^ comes from the vibrations of Si-O-(Si) and Si-O-(Al) bonds, which are probably related to the presence in the structure of zeolite as tetrahedrons, a building unit material [15,16].

In the case of the samples after modification with metals (Figure 7b,c), regardless of the metal used or the modification of the initial material with ammonium exchange or its omission, it is visible that the bands in the range of 3600–3200 cm^−1^ are much less intense than in the case of the initial material. It can also be noticed that the band at 1650 cm^−1^ is less visible than in the case of ground-CLI. This may mean that the materials have been properly dried and the zeolite water content is much lower. An intense band between 1200 and 850 cm^−1^ can also be noticed. This is confirmed by the presence of vibrating Si–O (Si) and Si–O (Al) bonds [15].

**Figure 8:**
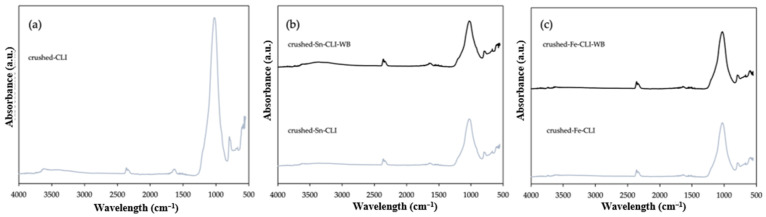
ATR FTIR spectra of crushed clinoptilolite without modification (**a**) and after modification with Sn metal (**b**) and Fe metal (**c**).

Figure 8a shows the FTIR analysis for the initial sample—clinoptilolite, properly prepared to a grain size of 0.2 mm by crushing larger forms of deposits and its modification by ammonium exchange and the introduction of the metals tin (Figure 8b) and iron (Figure 8c). Clinoptilolite was also modified with metals without ammonium exchanged. Based on the collected results, it is possible to notice characteristic bands that also occur for ground and metal-modified material with and without ammonium exchange (Figure 7). The only difference that can be observed is the lack of a band at 1600 cm^−1^, which indicates the presence of zeolite water [15]. However, a band of 2400–2300 cm^−1^ appears, which is responsible for the presence of CO_2_, but it does not affect the structure of the materials [17].

**Figure 9:**
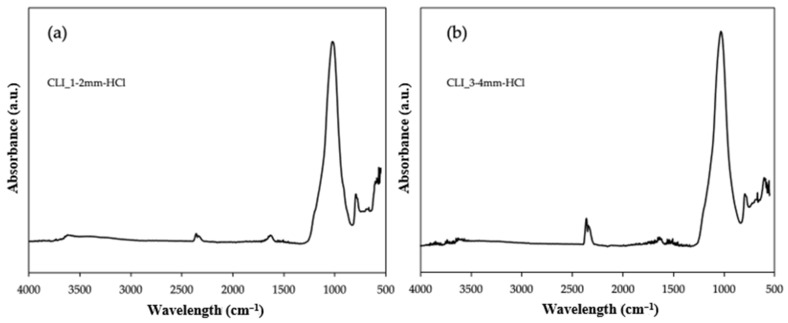
ATR FTIR spectra of clinoptilolite after hierarchization with hydrochloric acid. (**a**) Size 1–2 mm and (**b**) size 3–4 mm.

Additionally, FTIR analysis was performed for samples after hierarchization with hydrochloric acid. Figure 9 shows the results for clinoptilolite with a fraction of 1–2 mm (Figure 9a) and with a fraction of 3–4 mm (Figure 9b). Comparing the obtained spectra, it can be seen that they do not differ compared to samples with smaller grain sizes or modified by ammonium exchange or metals (Figure 7 and Figure 8). However, it can notice the presence of a band at 2400–2300 cm^−1^, which is responsible for the presence of CO_2_ [17]. However, this peak is more visible for the clinoptilolite sample after hierarchization with a larger fraction of 3–4 mm. This may be related to the short degassing time of the sample before analysis. However, the presence of gas does not affect the structure of clinoptilolite. Additionally, zeolite water is present in both samples and has a peak at 1650–1600 cm^−1^ [15]. Both samples were compared with clinoptilolite with a fraction of up to 0.2 mm, prepared by crushing, and a characteristic band at 1200–9000 cm^−1^ for samples after hierarchization, and precisely the peak of this band was shifted towards higher wavelengths. This may indicate that aluminum has been removed from the structure [18].

**Figure 10:**
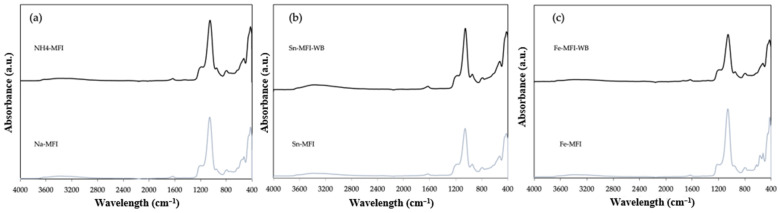
ATR FTIR spectra of zeolite: (**a**) sodium form and after ammonium exchange (**b**) with incorporated Sn metal, (**c**) with incorporated Fe metal.

In the case of the synthetic counterpart, FTIR analyses were also performed for the starting material and the ammonium-exchanged material (Figure 10). Additionally, the results for zeolite after modification with metals with and without ammonium exchange were also presented. Comparing the results with natural counterparts, it can be seen that there are characteristic peaks at a wavelength of 3600–3400 cm^−1^, which correspond to the presence of the -OH group, and a small band at 1650–1600 cm^−1^ can be seen, which confirms the presence of zeolite water. A spreading intense peak in the range of 1200–900 cm^−1^ corresponds to the vibrations of Si-O-(Si) and Si-O-(Al) bonds, which are probably related to the presence in the structure of zeolite [15,16].

Table 3 shows most of the analyzed materials with a fraction of up to 0.2 mm for natural and synthetic zeolite. It can be seen that, in the case of the starting CLI material, the specific surface area is 29 m^2^/g. After its modification by ammonium exchange, there was only a slight increase to 30 m^2^/g. In turn, further modifications with metals show a significant increase in the specific surface area. In the case of iron modification, the specific surface area increased to 85 m^2^/g for ground-Fe-CLI and up to 165 m^2^/g for ground-Sn-CLI. Such a visible increase in specific surface area is probably related to metals not blocking access to the pores for the analyzed nitrogen, and with a high probability that agglomerates in the form of metallic oxide forms were formed on the surface of the tested material, which, as -O-M-O- oligomers, could significantly increase the specific surface area of the materials [18]. Modification of clinoptilolite with metals and earlier ammonium exchange resulted in a significant increase in the specific surface area of the samples. The layered structure of zeolite also has an influence here, where modifications could increase the distance between the plates in the zeolite and ammonium exchange allowed for the washing out of alkaline cations and other crystalline phases, such as quartz or illite. Removal of alkali metal cations and other crystalline phases and amorphous residues probably unblocked access to pores, including micropores, making nitrogen diffusion during BET analysis much easier and much more nitrogen being adsorbed, resulting in an increase in the specific surface area of the materials [18]. The elution of other crystalline phases can be observed in the diffractograms (Figure 3 and Figure 4) of samples after ammonium exchange and the introduction of tin and iron.

In turn, in the case of synthetic zeolite MFI, the situation is the opposite. Such results are probably influenced by the microporous structure of this material. During metal modification, regardless of the MFI modification method, some metals are located in zeolite pores, which may block nitrogen access during analysis. It can also be noted that ammonium exchange and then ion exchange to deposit specific metals is a better modification because the specific surface areas are much higher than when metals are directly introduced without the hydrogen form [19].

**Figure 11:**
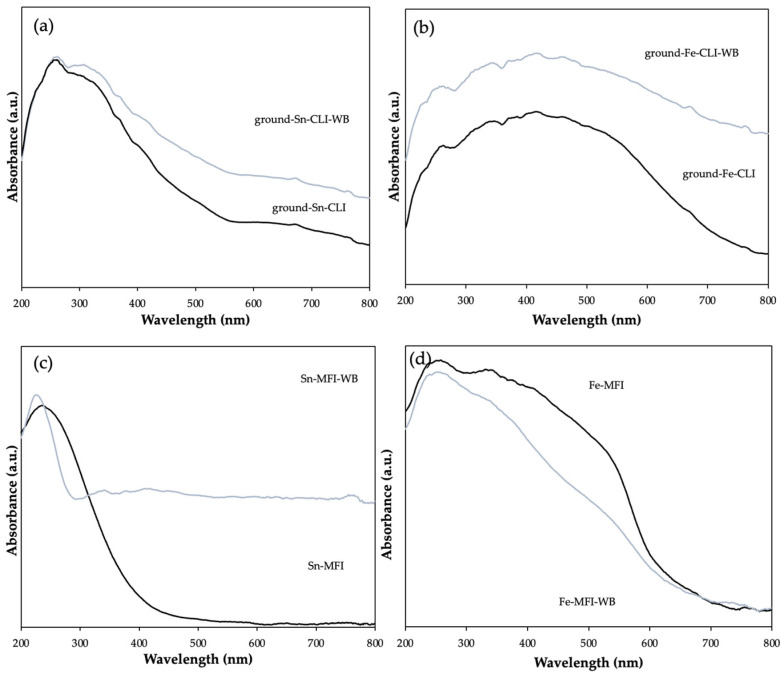
DRS UV-Vis spectra of clinoptilolite. (**a**) Modification with Sn metal and (**b**) Fe metal and MFI. (**c**) Modification with Sn metal and (**d**) Fe metal.

Figure 11 shows the results of the DRS UV-Vis analysis for natural and synthetic zeolite modified with tin and iron. The analysis was performed to provide information on the available metals and in what form they occur. First, you can see the difference in the absorption intensity bands of the tested samples. Higher intensities may indicate the presence of zeolite water, which affects the performance of analyses, and in the case of natural zeolite, the presence of other crystalline phases and metals such as calcium or potassium may also be responsible for such a difference. However, according to the literature, it can be noticed that at 250–300 nm for samples regardless of the material used, a metal, tin, in the oxide form SnO_2_ is present [18,19]. In turn, for iron, the wavelength between 230 and 250 nm is responsible for the presence of iron in the form of Fe(III) [20].

In order to determine the elemental composition, an analysis was performed using an EDS detector. The composition analysis was performed for natural zeolite after grinding and crushing, and the Sn and Fe content was checked after ion exchange with and without ammonium exchange. Additionally, analyses were performed for the starting synthetic zeolite MFI, then after ammonium exchange, and the presence of metals was checked after further modifications with and without ammonium exchange.

**Figure 12:**
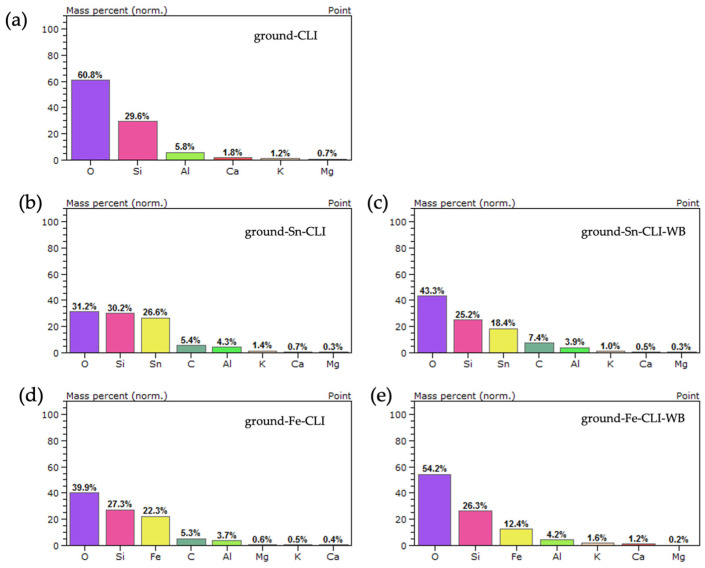
EDS analysis for ground zeolite: (**a**) without modification, (**b**) after ammonium exchange and Sn modification, (**c**) without ammonium exchange and Sn modification, (**d**) after ammonium exchange and Fe modification, (**e**) without ammonium exchange and modification Fe.

Based on the analysis performed (Figure 12), the presence of silicon, aluminum and oxygen, which constitute the zeolite skeleton, can be observed for the initial sample (ground-CLI). Additionally, alkali metals such as calcium, potassium and magnesium were observed. In the case of the sample after ammonium exchange and modification with tin, its presence was noted at a level of 26.6 wt.%. Additionally, there was a drop in the aluminum content of 4.3 wt.% and alkali metals at the analyzed point. The same situation applies to the sample without ammonium exchange after modification with tin. The presence of tin at the point site was observed at 18.4 wt.%, along with a decrease in alkali metals and aluminum (3.9 wt.%). In the case of samples with iron, spot analysis also observed the presence of the metal at a level of 22.3 wt.% (ground-Fe-CLI) and 12.4 wt.% (ground-Fe-CLI-WB).

**Figure 13:**
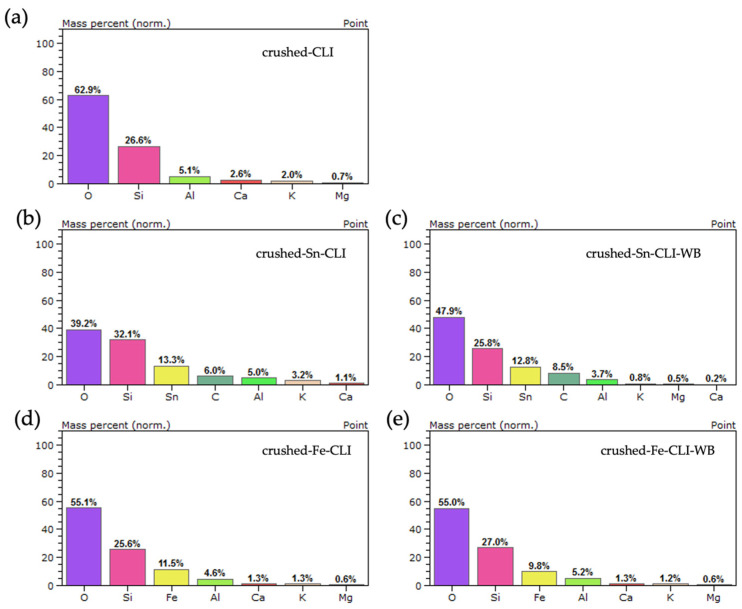
EDS analysis for crushed zeolite: (**a**) without modification, (**b**) after ammonium exchange and Sn modification, (**c**) without ammonium exchange and Sn modification, (**d**) after ammonium exchange and Fe modification, (**e**) without ammonium exchange and modification Fe.

The Figure 13 presents the results of the EDS analysis for clinoptilolite prepared by crushing larger forms to a grain size of 0.2 mm. It can be seen that in the case of the material without modification, silicon, aluminum and oxygen were observed, which are responsible for the zeolite skeleton. Additionally, the same alkali metals were also observed as in the case of ground clinoptilolite (Figure 12). In the case of further modifications with metals, their presence at the analyzed point can be noticed. In both cases, the metal content is higher after ammonium exchange: 13.3 wt.% for crushed-Sn-CLI and 11.5 wt.% for crushed-Fe-CLI. It can also be noticed that the mas% content for alkali metals is lower than in the case of the starting material, which may indicate its leaching during zeolite modification.

**Figure 14:**
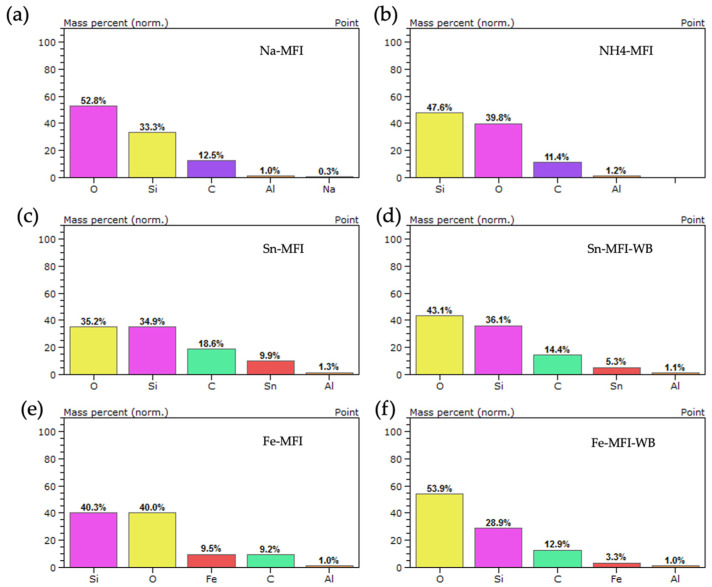
EDS analysis for MFI zeolite: (**a**) without modification—sodium form, (**b**) after ammonium exchange, (**c**) after ammonium exchange and Sn modification, (**d**) without ammonium exchange and modification Sn, (**e**) after ammonium exchange and modification Fe, (**f**) without ammonium exchange and modification Fe.

A point analysis of the elemental composition was also performed for the synthetic MFI zeolite (Figure 14) without modification and after ammonium exchange. It can be seen that the starting material contains sodium, which is removed after ammonium exchange. In the case of metal modification, in each case regardless of the previous ammonium exchange or its absence, the presence of individual metals with a mass content of 9.9 wt.% for Sn-MFI, 5.3 wt.% for Sn-MFI-WB and 9.5 wt.% for Fe was observed, respectively, -MFI and 3.3 wt.% for Fe-MFI-WB. The presence of sodium was not observed after further modifications, which indicates well-selected ammonium exchange conditions.

### 3.2. Zeolites Adsorption Tests

For all adsorbents tested, adsorption measurements with test odorants (e.g., acetaldehyde, dimethylamine, propionic acid, pentanoic acid and octanoic acid) (Merck KGaA, Darmstadt, Germany) were performed in the liquid phase using thermostated quartz reactors with a magnetic stirring system and in the gas phase on an adsorption test rig equipped with a tubular reactor with a fixed adsorbent bed coupled to a chromatographic measurement line. The liquid supernatant was analyzed using an Agilent 6890N (Santa Clara, CA, USA) gas chromatograph (GC) with a BP21 FFAP capillary column (60 m, 0.25 mm diameter) and a flame ionization detector (FID). For dimethylamine analysis, an HP 5890 series II gas chromatograph (Hewlett Packard Company, Palo Alto, CA, USA) with an HP-5 capillary column (30 m, 0.24 mm diameter) and NPD (nitrogen–phosphorus detector) was used.

The adsorption capacity studies were performed in two phases: liquid and gas. The description of the process conditions is presented in Table 4.

**Figure 15:**
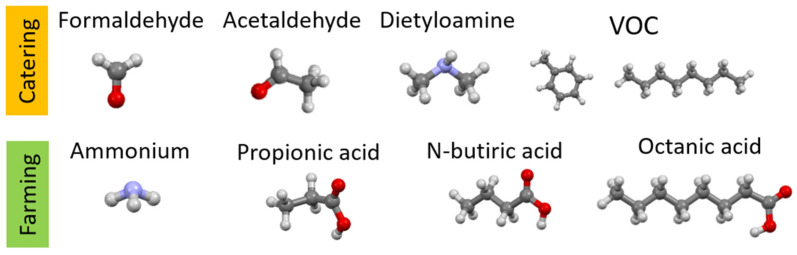
Selected odor compounds found in gastronomy and culture—for screening tests of the adsorption properties of zeolite materials. Elements color: red—oxygen, light blue—nitrogen, dark gray—carbon, lighter gray—hydrogen.

Taking into account the chemical similarity of odorous substances, their occurrence in various branches of human activity (e.g., catering, farming) and the effective possibility of carrying out studies on a large number of modified natural zeolites in the laboratory, as well as the time frame of the project, the following group of odorous compounds was selected for laboratory studies (Figure 15): acetaldehyde, dimethylamine, propionic acid, pentanoic acid (otherwise known as valeric acid) and octanoic acid. This is due to both the time frame of the project as well as the chemical similarity of the odorous substances. Similar substances, e.g., acids, will require the presence of analogous adsorption centers. Another important parameter will be the size of the molecules, hence the selection of, e.g., acids with different carbon chain lengths (e.g., propionic acid, octanoic acid) to investigate the role of chain length on the adsorption process [11].

In the case of formaldehyde and acetaldehyde, the adsorption process will take place on the same type of zeolite centers; so, due to a more convenient selection of process parameters during laboratory work, acetaldehyde was selected for adsorption studies. For nitrogen-containing compounds, dimethylamine can be mentioned. For chemical reasons, they will adsorb on similar active centers. However, it should be noted that dimethylamine in a mixture with oxygen is highly explosive, so it can be tested in liquid phase in the range of applied concentrations.

#### 3.2.1. Adsorption of Acetaldehyde (AD)

First, the adsorption process of acetaldehyde was studied for a series of zeolites with different degrees of modification. In the study of acetaldehyde adsorption in the liquid phase, about 20% of acetaldehyde solution dissolved in 20 mL of ethanol was used per 1 g of zeolite. The experiments were carried out at three temperatures: 25, 40 and 60 °C.

**Figure 16:**
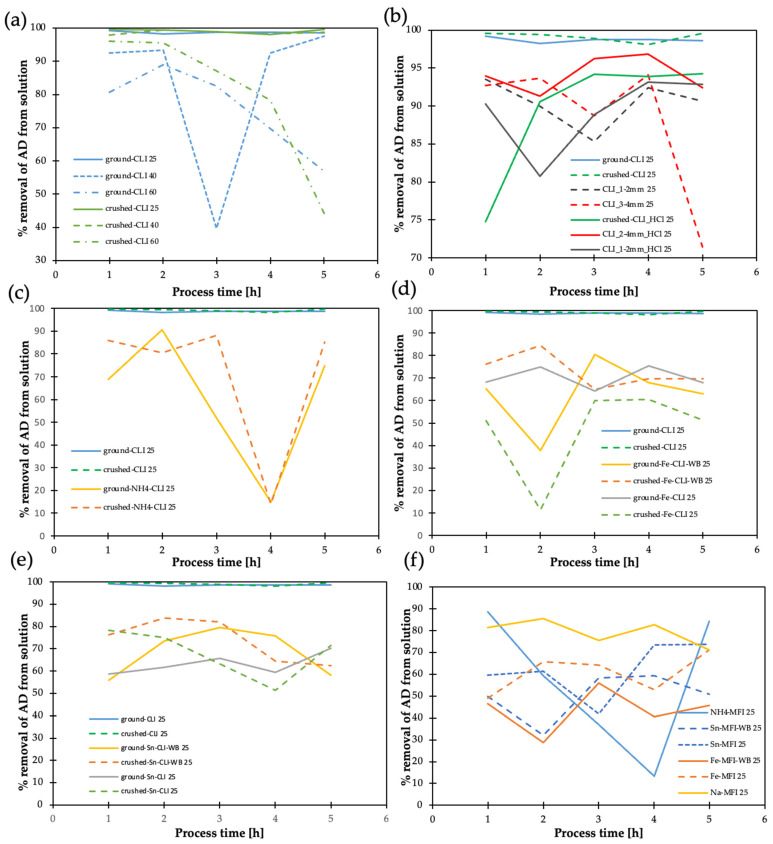
Result of acetaldehyde adsorption efficiency on clinoptilolite (different fractions) and synthetic zeolite (MFI): (**a**) crushed and ground, 25, 40, 60 (temperatures), (**b**) comparison of different fractions of natural and hierarchical clinoptilolite, (**c**) crushed and ground clinoptilolite—ammonium version, (**d**) with and without direct exchange—iron, (**e**) with and without direct exchange—tin, (**f**) synthetic zeolite MFI with listed metals.

In the case of clinoptilolite ground and crushed, very good adsorption results (more than 95%) were obtained at 25 °C (Figure 16a), while at higher temperatures, a high variability of adsorption is observed, leading to the conclusion that it will be most beneficial to carry out the process in the liquid phase at room temperature (25 °C).

Hierarchization of CLI 3–4 mm with hydrochloric acid has a favorable effect on AD removal efficiency (~95%) (Figure 16b). In contrast, the ammonium form of the zeolite is unfavorable for the AD adsorption process: it is removed with much lower (by 20–70%) efficiency in the presence of the ammonium form and the process is unstable over time for the ammonium form (Figure 16c). The introduction of Sn and Fe is unfavorable for the AD adsorption process (Figure 16d,e): it is removed with much lower (by 20–50%) efficiency in the presence of these metals and unfavorable adsorption processes are observed, which can lead to the formation of more harmful compounds. Synthetic zeolite (Figure 16f) was found to have 10–20% worse AD adsorption capacity and the introduction of Sn and Fe into the synthetic zeolites worsens the adsorption process even further.

#### 3.2.2. Adsorption of Dimethylamine (DMA)

**Figure 17:**
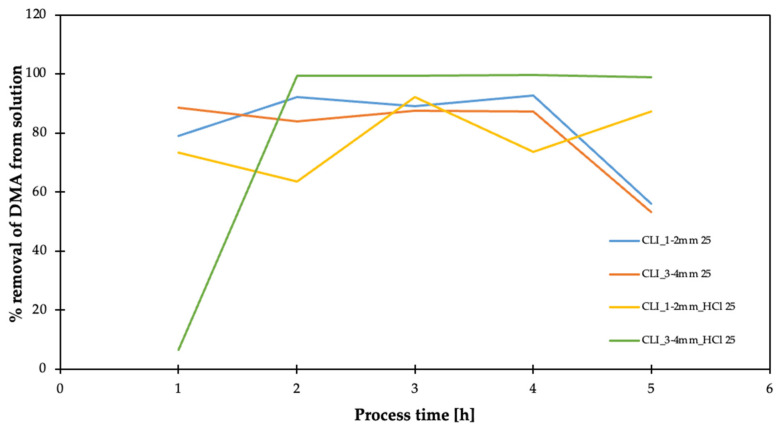
Result of adsorption efficiency of dimethylamine on clinoptilolite.

The 1–2 mm and 3–4 mm CLI fractions were considered for DMA (Figure 17). In the liquid phase, dimethylamine adsorption studies of 5% dimethylamine solution dissolved in 10 mL of ethanol was used per 1 g of zeolite. The experiments were carried out at 25 °C.

They have sufficient stability of dimethylamine removal. In addition, the hierarchization of the 3–4 mm fraction initially results in no DMA removal, but has very high DMA removal efficiency after prolonged operation.

#### 3.2.3. Adsorption of Propionic Acid (PA), Pentanoic Acid (VA) and Octanoic Acid (OA)

**Figure 18:**
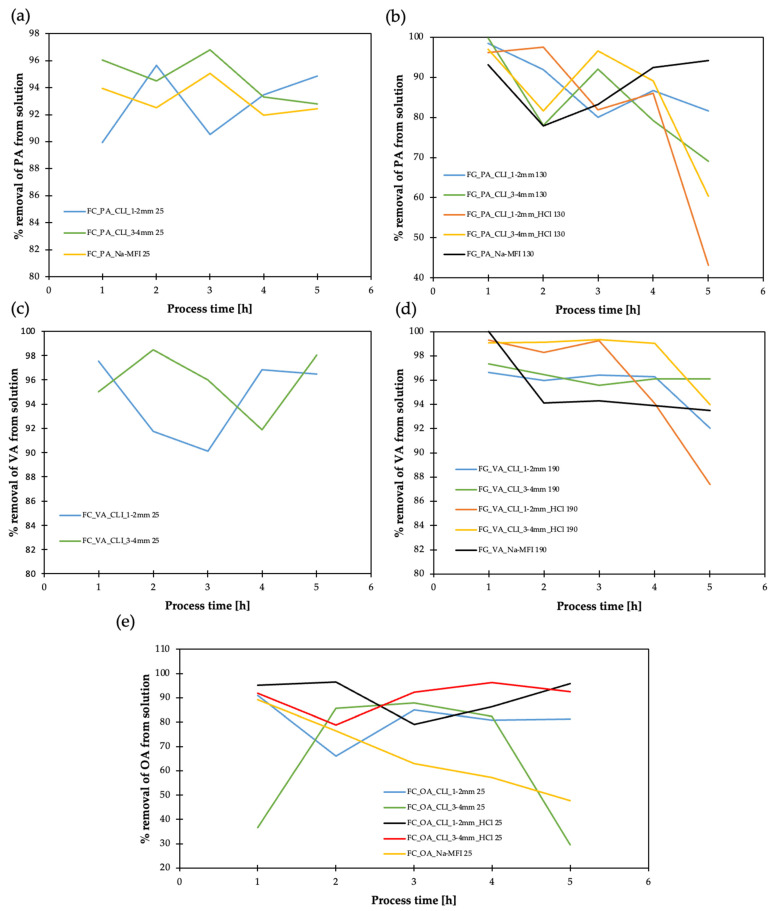
Result of adsorption efficiency of organic acids on clinoptilolite and synthetic zeolite (MFI): (**a**) propionic acid in liquid phase (FC), (**b**) propionic acid in gas phase (FG), (**c**) pentanoic acid in liquid phase (FC), (**d**) pentanoic acid in gas phase (FG), (**e**) octanoic acid in liquid phase (FC). Sample codes for zeolites: CLI—clinoptilolite, 1–2 and 3–4 mm—fractions, HCl—HCl-hierarchized zeolite, MFI—synthetic zeolite type ZSM-5, Na—sodium form, NH_4_—ammonium version. Odorant codes: PA—propionic acid, VA—pentanoic acid, OA—octanoic acid, 25, 130, 190—temperatures.

Selected carboxylic acids were adsorbed: propionic acid (PA), pentanoic acid (VA) and octanoic acid (OA) (Figure 18). The carboxylic acid adsorption tests were carried out on selected zeolite materials in the liquid and gas phase. In the case of the liquid phase adsorption process, all three carboxylic acids were used. The adsorption conditions in the gas phase were developed as follows: adsorption time process 5 h, temperature 25 °C, 1 g of zeolite material and 10 mL of ethanol 5% solution of propionic, pentanoic and octanoic acids. Samples were taken every hour and analyzed with a gas chromatograph with FID detector. A gas-phase adsorption process was also carried out using various zeolite materials. In this case, propionic acid and pentanoic acid were used. The conditions developed for the adsorption process are as follows: process adsorption—5 h, the amount of zeolite material used—1 g, 10 mL water solutions of 5% propionic acid and pentanoic acid. The process temperature for PA was 130 °C and for VA was 190 °C. Samples were collected every hour during the process and analyzed with a gas chromatograph with FID detector.

By analyzing the adsorption efficiency results, it can be concluded that the 3–4 mm CLI fraction is more favorable for PA in the liquid (Figure 18a) and gas (Figure 18b) phase. Additionally, adsorption with CLI of 3–4 mm fraction is noticeable for reducing the amount of pentanoic acid (VA). In this case, adsorption occurs better in the liquid phase (Figure 18c). Hierarchization is not very important in this case for PA and VA adsorption. However, hierarchization of clinoptilolite, CLI 3–4 mm, with hydrochloric acid has a favorable effect on the removal efficiency of OA (~95%) (Figure 18e), which is reasonable due to the long length of octanoic acid. Similar to AD, the ammonium form of CLI is unfavorable for the adsorption of the studied acids.

### 3.3. Theoretical Modelling

As part of the publication, calculations using the DFT method were also performed. For this purpose, a cluster model of zeolites was developed and computational processes for adsorption were performed [21,22,23].

The crystal structure of zeolite, clinoptilolite, was collected from the available database of the International Zeolite Association (IZA) [24]. The electronic structure of all clusters was calculated in the StoBe program [25] using the non-local generalized gradient corrected functionals according to Perdew, Burke and Ernzerhof (RPBE) [26,27,28], in order to account for electron exchange and correlation. A double zeta valence polarization (DZVP) type was used for the orbital basis sets of Sn (633321/53321/531), Co, Cu and Fe (63321/531/311), Si (6321/521/1), Al (6321/521/1), O, C (621/41/1) and H (41). Auxiliary basis sets, such as (5,5;5,5) for Si, Sn, Fe, Cu and Co (4,3:4,3) for O, C, N and (41) for H, were applied to fit the electron density and the exchange-correlation potential. The methodology is similar to that published in our previous work on the development of BEA and CLI type zeolite clusters [17].

**Figure 19:**
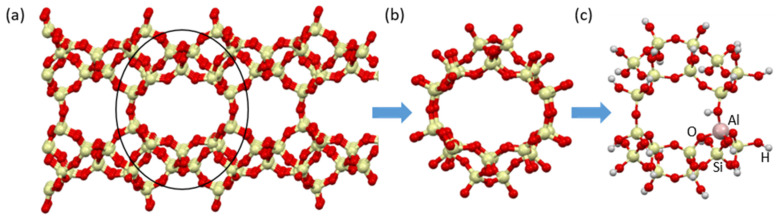
Clusters used for the CLI: (**a**) bulk structure, (**b**) hierarchical structure, (**c**) cluster model with exchanged Al.

Several clusters representing CLI zeolite were analyzed. Finally, the following were used in the adsorption studies: AlSi_17_O_50_H_29_ for CLI (ideal form) (Figure 19). Taking into account the experimental results and the unfavorable influence of synthetic zeolites on the odorant adsorption process, the theoretical modeling process focused on the study of natural clinoptilolite. The crystal structure of clinoptilolite was reconstructed (Figure 19a) and representative cluster models of clinoptilolite were prepared: (i) a supercell model with a size of about 12 Å (Figure 19b), (ii) a supercell fragment model (to reduce the CPU time for the adsorption process, Figure 19c). Broken bonds were saturated with hydrogen atoms. The geometry of all investigated structures was optimized. Subsequently, some Si cents were exchanged for Al and the presence of Brønsted-type -OH acid centers near the Al centers were taken into account in order to maintain an appropriate lattice charge.

#### 3.3.1. Modeling the Odors Adsorption Process

**Figure 20:**
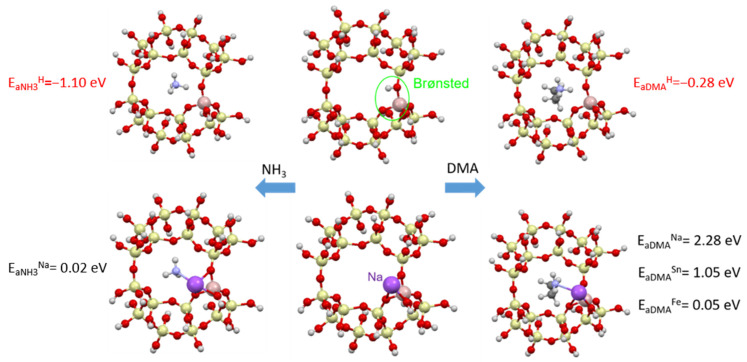
Adsorption of ammonia (**left column**) and dimethylamine (**right column**) in a clinoptilolite chamber (**middle column**): (**top**) adsorption on Brønsted acid centers, (**bottom**) adsorption on metallic centers (Na, Sn or Fe).

The adsorption of ammonia and dimethylamine in a clinoptilolite supercell in the presence of Brønsted centers and metals (Na, Fe and Sn) was modeled. Figure 20 shows the adsorption energies of NH_3_ and DMA and the optimized zeolite-adsorbant structures.

For both odorants containing nitrogen, stable adsorption (with adsorption energy E_a_ < 0) is observed in the presence of Brønsted acid centers (Figure 20). In the presence of metals, the adsorption process will not be stable (E_a_ > 0), which agrees with the experimental data obtained, where the addition of metals worsens the adsorption process.

**Figure 21:**
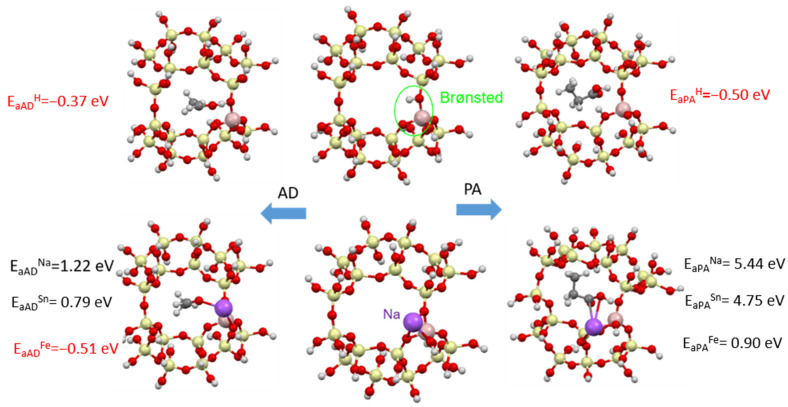
Adsorption of acetaldehyde (**left column**) and propionic acid (**right column**) in a clinoptilolite chamber (**middle column**): (**top**) adsorption on Brønsted acid centers, (**bottom**) adsorption on metallic centers (Na, Sn or Fe).

For acetaldehyde and propionic acid, stable adsorption (with adsorption energy E_a_ < 0) is observed when Brønsted acid centers are present (Figure 21). In addition, for acetaldehyde, the addition of iron, which by the way is present in small amounts in natural clinoptilolite, is also favorable. In the presence of metals for acids, the adsorption process will not be stable (E_a_ > 0), which agrees with the experimental data obtained, where the addition of metals worsens the adsorption process.

##### Summary

- Stabilization of all groups of odorous substances on clinoptilolite supercell walls occurs near Brønsted-type acid centers (-Al-OH), so increasing the number of acid centers will increase the sorption properties of clinoptilolite (E_a_ < 0)
- The presence of alkali metals and transition metals (Sn or Fe) does not favor the adsorption of most odorous substances (E_a_ > 0)
- It is possible to trap long odorants (e.g., octanoic acid with a chain length of 11.1 Å) in clinoptilolite supercells (about 12 Å in size).

## 4. Conclusions

In conclusion, dried natural zeolites of different granulation (clinoptilolite, CLI) and using different binders and synthetic zeolite, which were subjected to different modifications (alkali metals leaching, metal, Sn and Fe subsidization, hierarchization in acidic medium), were investigated, and the adsorption process of selected odorants (acetaldehyde, dimethylamine, propionic, pentanoic and octanoic acids) was studied.

The possibility of using clinoptilolite with a larger granulation (above 2 mm) as an adsorbent is important due to the economics of production of anti-odor filters, and the temperature stability of the odor adsorption process compared to the metal-modified material makes it advisable to use this particular material in actual anti-odor filters.

A summary of adsorption efficiency is as follows:

- A fraction of 3–4 mm CLI most favorable for removal of VA and PA.
- Hierarchization of 3–4 mm CLI with hydrochloric acid is favorable for the removal efficiency of AD and OA only.
- Fractions of 1–2 mm and 3–4 mm CLI have sufficient stability for dimethylamine removal.
- Transition metals and modified synthetic zeolite should absolutely not be introduced into the zeolite mixture for odor adsorption purposes. This is because they deteriorate the removal efficiency of most of the tested odorous substances and may cause unfavorable processes (e.g., catalytic), which may lead to the formation of new unfavorable substances (including odorous substances).
- The use of a fine clinoptilolite fraction (less than 0.2 mm, 80%) with the addition of aluminosilicate-based binders (20%) granulated to a grain size of 3–5 mm shows promising adsorption properties. When choosing between pure clinoptilolite and granule fractions, economic factors (price of formulation) should be taken into account, as there is little difference in efficiency of 5–10% for AD adsorption.

Modification of zeolite CLI and MFI with metals does not improve the adsorption capacity of these materials in odor removal. The reason is that metals are present in the form with the highest oxidation state and only its activation at a higher temperature can lead to a decrease in the oxidation degree, i.e., such materials behave like catalysts, not adsorbents. If activated, sorption materials could lead to uncontrolled chemical reactions rather than adsorption and trapping of chemicals. Another important issue is that the adsorption material should have a Brønsted center in its structure, which may constitute a site for the adsorption of chemical compounds and their adsorption. Therefore, zeolites may be promising materials that will trap chemical compounds, including unpleasant odors. The selection of zeolite is important because, due to the size of the adsorbed molecule, the size of the pores together with the presence of Brønsted active centers will be responsible for the adsorption of these compounds. Clinoptilolite can fulfill both functions because it is a mesoporous material containing pores of various sizes. Additionally, they contain the so-called super-chambers that can absorb larger molecules like octanoic acid. An important issue is the modification of zeolites to remove alkaline cations and introduce ammonium ions, which will be removed in the form of ammonia under the influence of temperature, obtaining a hydrogen form—beneficial for obtaining Brønsted centers and improving adsorption properties. Additionally, hierarchization using acids will also lead to obtaining a hydrogen form with the additional removal of other crystalline or alkali metal phases that block access to the pores.

In the case of acetaldehyde adsorption, the optimal material for its adsorption is clinoptilolite in powder form. The grain size has a significant impact due to the fact that the aldehyde has a low boiling point and changes its state to gas already at room temperature. The larger zeolite grains do not improve the adsorption properties and the AD molecule is easily desorbed. The possibility of adsorption of acetaldehyde is possible due to the presence of the hydrogen form of zeolite, which combines with oxygen from the aldehyde group. In the case of dimethylamine, zeolite has a larger grain size and, after hierarchization, is a better sorption material. This may be due to the presence of free skeletal oxygen in the anionic form, which combines with the formed cationic form of DMA. In the case of carboxylic acids, regardless of the length of the carbon chain, each of them adsorbs on material with a grain size above 1 nm and after modification with hydrochloric acid. Here, adsorption is possible due to the formation of a hydrogen form in the zeolite and an anionic form of carboxylic acid (-COO^−^).

## Figures and Tables

**Table 1:** Odorous substances found in catering establishments and their maximum allowed concentrations [8,9,10].

Pollutant	Source of Emissions	MCL [mg/m^3^]
Formaldehyde	Grills	0.5
NO_2_	Gas cookers and burners	0.7
CO	Cookers and burners. Fireplaces	25
CO_2_	Cookers and burners. Fireplaces	9000
PAH	Wood and coal burning	0.002
HAA	Frying, toasting, grilling meat	0.002
Acroleine	Frying in fat	0.05
Acetaldehyde	Baking	5
VOC	Cooking	0.7
Dimetyloamine	Grill and grill cleaner	9

MCL—maximum concentration limit. Set by the United States Environmental Protection Agency.

**Table 2:** Vent gas pollutant concentrations from piggery [11].

Pollution	Concentration [mg/m^3^]
Ammonia	18
Hydrogen sulfide	0.004
Skatol	0.003
Indole	0.003
Phenol	0.005
p-Cresol	0.04
Acetic acid	6.7
Propionic acid	1.1
N-Butyric acid	0.7
Isobutyric acid	0.16
N-Valeric acid	0.08
Isovaleric acid	0.21
N-caproic acid	0.01
Isocaproic acid	0.004
Heptanoic acid	0.003
Octanoic acid	0.005
Pelargonic acid	0.004

**Table 3:** Specific surface area and pore size of CLI and MFI zeolite.

Material	S_BET_ [m^2^/g]	S_BJH_ [m^2^/g]	V_p total BET_ [cm^3^/g]	V_p mikro_ [cm^3^/g]
CLI	29	16	0.06	0.006
ground-NH_4_-CLI	30	17	0.07	0.006
ground-Sn-CLI	165	110	0.15	0.025
ground-Fe-CLI	85	42	0.11	0.02
Na-MFI	396	209	0.56	0.116
Sn-MFI	368	187	0.55	0.099
Sn-MFI-WB	314	178	0.53	0.074
Fe-MFI	311	132	0.53	0.097
Fe-MFI-WB	284	121	0.23	0.09

**Table 4:** Description of odor adsorption processes by phase and number of samples taken and time of chromatographic analyses performed.

Adsorption in the Liquid Phase	Adsorption in the Gas Phase
The process conditions were established and standardized for all odorants: duration (5 h), temperature (25–60 °C), solvent (ethanol), adsorbent mass (1 g). Every hour, samples are taken to measure the adsorption capacity. The apparatus does not require continuous supervision. For 1 zeolite + 1 odorant sample, a time of 1–5 h is allocated. Then, collected samples are subjected to gas chromatographic analysis.	The reaction charge is, for example, a 5% solution of odorant at a flow rate of 2 mL/h, injected by an infusion pump. The adsorbant then enters a mixer where it mixes with the inert gas (nitrogen) and is converted to the gas phase. Driven by the gas stream (nitrogen, 50 mL/min), the odorant vapors wash over the adsorbent bed (mass 0.5 g), where the adsorption process takes place. Then, collected samples are subjected to gas chromatographic analysis.

## Data Availability

The original contributions presented in the study are included in the article, further inquiries can be directed to the corresponding author.
